# Cardiac abnormalities in Anderson-Fabry disease and Fabry’s cardiomyopathy

**Published:** 2011-02

**Authors:** Ryan P Morrissey, Kiran J Philip, Ernst R Schwarz

**Affiliations:** Division of Cardiology, Department of Medicine, Cedars-Sinai Medical Centre and David Geffen School of Medicine at UCLA, Los Angeles, California, USA; Division of Cardiology, Department of Medicine, Cedars-Sinai Medical Centre and David Geffen School of Medicine at UCLA, Los Angeles, California, USA; Division of Cardiology, Department of Medicine, Cedars-Sinai Medical Centre and David Geffen School of Medicine at UCLA, Los Angeles, California, USA

**Keywords:** Fabry’s, Anderson-Fabry, left ventricular hypertrophy, restrictive cardiomyopathy, tissue Doppler imaging, cardiac MRI (CMR), late/delayed gadolinium enhancement

## Abstract

**Summary:**

Fabry’s disease is an X-linked lysosomal storage disease most often associated with renal dysfunction and death due to renal failure in patients’ fourth and fifth decades of life. However, cardiac manifestations including arrhythmias, angina and heart failure are common and probably under-recognised. Furthermore, Fabry’s disease is now recognised as also affecting female carriers, who manifest signs later than males. A variant of Fabry’s has been identified that only affects cardiac tissue, which presents as an unexplained hypertrophy of the left ventricle in middle-aged patients, possibly with women more affected than men.

Given that epidemiological studies report a prevalence of Fabry’s cardiomyopathy among middle-aged patients with cardiac hypertrophy to be anywhere from one to 12%, it is reasonable to screen these patients for alpha-galactosidase A deficiency. Although mortality data is lacking from randomised, controlled trials of galactosidase replacement therapy, there are some reports of improvement in cardiac endpoints. Therefore patients with known Fabry’s disease should be screened early for cardiac involvement, as treatment benefit may not be seen once cardiac fibrosis has developed.

## Summary

Fabry’s disease is one of roughly 40 lysosomal storage disorders that result in the accumulation of glycoproteins. Also called Anderson-Fabry disease, Fabry’s is caused by mutations in the GLA gene, which encodes alpha-galactosidase A, resulting in accumulation of glycosphingolipids, specifically globotria-oslyceramide, within the lysosomes.^1^ This accumulation leads to cellular dysfunction, particularly in the endothelium, resulting in hypo-perfusion of tissues and further inflammation. The most frequently involved organs include the kidneys, heart, peripheral nerves and skin.^2^ Although an X-linked condition, heterozygous females as well as hemizygous males can be affected.

Anderson-Fabry disease, or angiokeratoma corporis diffusum, was independently described by Johann Fabry in Germany and William Anderson in England in 1898 in separate dermatology journals.^3^,^4^ However, it was not until 1963 when it was classified as a storage disorder,^5^ and 1967 when the enzymatic defect was identified.^6^ More recently, an atypical variant of Fabry’s, which presents as cardiac hypertrophy in middle-aged adults has been identified.^7^

The incidence of Fabry’s disease is estimated to be 1:55 000 male births,^1^ however, due to the constellation of presenting symptoms as well as some mutations allowing limited alphagalactosidase A activity, the actual incidence of Fabry’s, including atypical, sub-clinical or late-variant phenotypes is likely to be much higher, even as high as 1:3 100 male births.^8^

Manifestations of Fabry’s disease are directly related to accumulated glycosphingolipids in tissues. Clinical symptoms usually present in childhood with painful neuropathy of the hands and feet, nausea and abdominal pain.^2^ Angiokeratomas, collections of enlarged cutaneous capillaries, specifically of the trunk, increase in number and size with age. Proteinuria and eventual renal failure result from the accumulation of glycosphingolipids in the tubular epithelial, glomerular and endothelial cells, resulting in both focal and diffuse glomerulosclerosis and microvascular dysfunction. Males progress to renal failure by their fourth decade and females by their fifth decade if untreated.^9^

Transient ischaemic attacks and strokes are 12 times more common than the general population in males between the ages of 25 and 44.^2^ Other neurological symptoms include decreased hearing, anhydrosis, abnormal peripheral sensation, e.g. temperature, pin-prick, and the neuropathic pain previously mentioned. Corneal opacities can also develop, although these usually do not affect vision. As will be discussed subsequently, manifestations of cardiac involvement include palpitations, dyspnoea or angina, usually secondary to an arrhythmia or myocardial hypertrophy.

Female ‘carriers’ or heterozygotes can also be affected and more often have cardiac manifestations.^10^ Signs of organ dysfunction due to glycosphingolipid accumulation also present later in women, with neurological features presenting at a mean age of 16 years and cardiac and renal involvement manifesting at mean ages of 33 and 37, respectively.^11^ The mechanism by which women are affected by this X-linked condition is most often attributed to X-inactivation; however, this has been called into question.^12^,^13^

One group reports the median cumulative age of death as being 50 years in males and 70 in females,^14^,^15^ although another series reports mean ages of 45 and 55 years, respectively.^16^ It has been generally stated in the literature that, at least prior to enzyme replacement therapy, men died from the consequences of renal failure, and women succumbed to cardiac complications.^2^,^14^-^17^

## Cardiac involvement

Fabry’s patients with cardiac involvement may present with dyspnoea, palpitations, syncope or angina, depending on the cardiac tissue involved. Classic Fabry’s is associated with cardiac manifestations including arrhythmias, valvular abnormalities and cardiomyopathy; however, the cardiac variant of Fabry’s presents later in life as left ventricular hypertrophy (LVH) with residual alpha-galactosidase A activity.^7^ Sixty per cent of Fabry’s patients have some cardiac manifestation, usually arrhythmias.^16^

As in other organ tissues, cardiac dysfunction is due to glycosphingolipid accumulation in the myocytes and conduction tissue, but probably more importantly with respect to the cardiomyopathy is myocyte hypertrophy and fibrosis as glycosphingolipid deposition accounts for less than 3% of the total myocardial mass.^18^ Hypertrophic myocytes contain vacuoles laden with sphingolipids, resulting in eventual fibrosis. The extent of myocyte hypertrophy and the accumulation of glycosphingolipid-laden vacuoles correlates with the extent of LV wall thickening on imaging.^19^ Similar cell degeneration occurs in valvular as well as conduction tissue.

## Cardiomyopathy

Although the hallmark of cardiac involvement in Fabry’s disease (classical or the cardiac variant) is LVH, Fabry’s cardiomyopathy is a restrictive cardiomyopathy,^20^-^22^ as it results from the accumulation of glycosphingolipids, whereas hypertrophic cardiomyopathy, i.e. non-physiological hypertrophy, is due to abnormal contractile proteins. However, the hallmark of restrictive cardiomyopathy is impaired ventricular filling, and in one series of 30 patients, no patient had severe diastolic dysfunction consistent with a restrictive pattern.^23^ Mild to moderate diastolic dysfunction is often present.^24^ Furthermore, most restrictive cardiomyopathies do not have LVH.

Amyloidosis can present with hypertrophy but it can easily be distinguished from Fabry’s in that the voltage is decreased on ECG. Also unlike other infiltrative cardiomyopathies, in Fabry’s only 1% of the myocardium contains the infiltrative material (glycosphingolipids). Therefore, as with other cardiomyopathies secondary to glycogen or lysosomal storage disorders,^13^ Fabry’s cardiomyopathy could be considered a ‘pseudo’-hypertrophic cardiomyopathy in that myocyte hypertrophy and fibrosis play a more prominent role than restrictive physiology in contributing to the heart failure.

In the cardiac variant of Fabry’s disease, patients have some alpha-galactosidase A activity and present later in life, often with only cardiac manifestations, although proteinuria can also be present.^7^ The cardiac variant, first described in 1990,^25^,^26^ may be due to alternative splicing in the alpha-galactosidase A gene,^27^ and in the first published case series, five of seven patients had no mutations in the coding regions despite very low levels of mRNA.^7^ The aetiology of the cardio-tropism of the cardiac variant of Fabry’s has not been elucidated.

Both the classical and cardiac variant of Fabry’s can cause global LVH or an asymmetrical septal hypertrophy similar to hypertrophic obstructive cardiomyopathy. However, systolic function is usually preserved, although there is a trend towards increased LV end-diastolic volume.^28^,^29^ Diastolic dysfunction is often present, but usually only moderate at worst and without a restrictive filling pattern.^23^,^24^ In heterozygous women with Fabry’s (mean age 40 years), 12.7% had concentric remodelling, 52.7% had concentric LV hypertrophy, and 10.9% had eccentric LVH by echocardiogram.^30^ Similar percentages were seen in hemizygous men, with 37% having concentric hypertrophy, and 37 and 10% having asymmetric septal hypertrophy.^23^

Hypertrophy is present in 51 to 55% of males with a median age of 43 to 45 years, versus 33 to 38% of females with a median age of 55 years.^18^,^31^ Gender, age and renal function are directly and independently related to the presence of LVH, but not to blood pressure, i.e. LVH is the direct result of myocardial infiltration and not from primary hypertension or secondary to renal dysfunction. The presence of LVH has also been shown to be logarithmically related to alpha-galactosidase A activity.^29^ In a relatively large case series of 1 448 Fabry’s patients, 11% of men and 6% of women had congestive heart failure (CHF) symptoms and 35% had evidence of cardiac hypertrophy. However, the mean ejection fraction (EF) was preserved (63.1 ± 9.1%).^32^

In a case-control series using echocardiography, tissue Doppler imaging (TDI) and cardiac MRI (CMR) to characterise Fabry’s cardiomyopathy, a distinct pattern of progression was seen in genetically- or biopsy-proven cases of Fabry’s disease.^33^ No patient had late gadolinium enhancement (LGE) (indicative of fibrosis, as will be discussed later) without LVH. Women younger than 20 years had no evidence of hypertrophy, normal radial and longitudinal function on TDI and no LGE. Women without LVH had reduced longitudinal function isolated to the lateral wall of the LV. Women with LVH had reduced longitudinal and radial function; women with LVH and LGE had severely reduced longitudinal and radial function.

Males without LVH had reduced longitudinal function in the lateral wall and septum, whereas males with LVH but without LGE also had reduced radial function. Males with LVH and LGE had severe longitudinal and radial dysfunction. In general, global LV function was not impaired.^33^ Similarly, another series found no evidence of either LVH or LV remodelling by echocardiogram in patients younger than 30 years old.^23^ Therefore, functional abnormalities arise before hypertrophy, and fibrosis visible on CMR only occurs after hypertrophy. These changes take time to develop as the glycosphingolipids accumulate.

The disproportionate amount of fibrosis in the lateral wall may be due to increased wall stress or a relatively oxygen-deficient environment. Lastly, there is no evidence of RV regional functional abnormalities or late-enhancement, possibly due to decreased work load of the right heart or that enhancement is more difficult to detect in the RV due to its smaller size and fewer myocytes.^33^

## Coronary artery disease/angina

Patients with Fabry’s have been documented as having an increased risk for coronary artery disease (CAD).^2^,^17^ However, in case-control series and cross-sectional studies, there is no evidence of increased admissions for myocardial infarctions, need for revascularisation procedures,^30^ or CAD by myocardial perfusion scan.^14^ However, there is a higher frequency of angina compared to case-matched controls and it is more common in patients with LVH.^30^ Angina is present in 13 to 20% of patients, and equal among men and women.^9^,^31^,^33^

This is not to say that patient’s with Fabry’s are not more susceptible to thrombotic or embolic phenomena. In one series of Japanese patients there was a high incidence of thrombotic events (9/65 with strokes).^34^ Despite a lack of significant coronary artery disease on angiograms, in one series, diffuse hypo-echogenic plaques were more common in Fabry’s patients compared to age-matched controls by intravascular ultrasound.^35^

It has also been shown that coronary flow is reduced in patients with Fabry’s despite normal peripheral endothelial function.^36^ However, patients with hypertrophic cardiomyopathy also have slow coronary flow compared to controls.^37^ Therefore the exact aetiology of angina in patients with Fabry’s is still open to debate.

## Arrhythmias

Patients with Fabry’s are more prone to both atrial and ventricular arrhythmias due to glycosphingolipid deposition and fibrosis as well as atrial dilatation and relative ischaemia secondary to LVH. However, in one series,^9^ both systolic and diastolic blood pressures were normal, implying that atrial arrhythmias, in particular atrial fibrillation, were not secondary to long-standing hypertension or renal disease but to Fabry’s. Interestingly, in this series arrhythmias were 1.5 times more likely in males (although other cardiac events were similar),^9^ possibly due to more advanced cardiomyopathy and renal dysfunction in hemizygotes versus heterozytgotes.

The most common ECG finding is voltage criteria for LVH,^13^ although various degrees of block as well as PR-interval shortening have also been described.^38^ Atrial arrhythmias, e.g. atrial fibrillation are more common than ventricular arrhythmias. In a case series of 78 patients over 10 years, 13% had paroxysmal atrial fibrillation (which was four times that of the general population for age) and 8% had non-sustained ventricular tachycardia (VT); all patients with VT had LVH. Predictors for atrial fibrillation were age, left atrial size, LV wall thickness, LV mass index and angina. Permanent pacemakers were implanted in 10.6% of patients for complete heart block or symptomatic bradycardia.^39^

An international series of 714 patients also found that the incidence of arrhythmias was increased in patients with LVH.^31^ Corresponding to observational reports that women die from cardiac aetiology, ventricular arrhythmias were more common in women, 20 versus 14%, in a large cohort of 1 448 patients.^32^

## Valvular disease

Mitral and aortic valve abnormalities were present in 57 and 47% of one series (where LVH was present in 61%), although no severe regurgitation was noted.^23^ Another larger series documents mitral valve regurgitation in 32% of patients.^32^ In a series of 111 patients, there were no cases of severe valvular disease, including nine patients with end-stage disease. The most common valvular abnormality was mild mitral regurgitation (*n* = 57).^40^ The incidence of Fabry’s patients undergoing valve replacement surgery is low.^31^ Therefore valves are not the most significantly affected tissue of the heart in patients with Fabry’s and do not cause much burden.

## Evaluating for cardiac involvement

In a retrospective cohort analysis of patients with amyloidosis, Fabry’s, hypertrophic obstructive cardiomyopathy and hypertensive heart disease patients, no single clinical characteristic, ECG finding or echocardiographic feature could differentiate between the various causes of LV hypertrophy, including echogenicity, valvular abnormalities, renal dysfunction and diastolic dysfunction. However, painful neuropathy, anhydrosis, lack of hypertension and presence of Sokolow-Lyon criteria for LVH on ECG were significant for Fabry’s disease by univariate analysis. Furthermore, if none of hypertension, orthostasis, pericardial effusion or a papillary muscle anomaly was present, the sensitivity and specificity for Fabry’s disease was 92 and 87%, respectively.^41^

## Echocardiography

As previously described, the echocardiogram shows varying degrees of hypertrophy and usually a preserved systolic function, with LVH more often seen in older individuals.^23^ Diastolic dysfunction, sometimes displaying a restrictive pattern, can be present,^23^ but peak E velocity, peak A velocity and deceleration time of the mitral valve are most often normal,^28^ and diastolic dysfunction is usually not present in the absence of LVH.^42^ Furthermore, diastolic dysfunction does not distinguish Fabry’s cardiomyopathy from hypertrophic obstructive cardiomyopathy.^43^ As previously discussed, valvular abnormalities, most often mitral and aortic, are not associated with severe regurgitation.^23^

A case-control series of 40 consecutive patients showed that 82.5% of Fabry’s patients – 94% of Fabry’s patients with LVH – had a ‘binary appearance’ of the endocardial border, which was not present in any matched hypertrophic cardiomyopthy patient, hypertensive or otherwise, representing a sensitivity and specificity of 94 and 100%, respectively, for detecting Fabry’s cardiomyopathy. Furthermore, pathological examination of the ‘binary’ areas revealed endocardium and myocardium laden with glycosphingolipids, separated by a subendocardial ‘empty space’.^43^

However, relying on the ‘binary sign’ has been challenged recently by Kounas *et al.*, where they found the sensitivity and specificity of the binary appearance on echocardiogram to differentiate Fabry’s from hypertrophic obstructive cardiomyopathy to be 35 and 79%, respectively. Furthermore, only 3.5% of patients with LV wall thickness less than 15 mm had a binary sign, albeit the number of subjects examined was small.^44^ A recent small, blinded study reported that a binary appearance of the endocardium on echocardiography has a sensitivity of only 15.4%, but a specificity of 73.3%.^45^

## Tissue Doppler imaging (TDI)

Although two-dimensional echocardiogram cannot be used to screen patients with Fabry’s disease for signs of cardiac involvement before the development of LVH, the addition of TDI may be reliable in detecting sub-clinical involvement. In a case-control series of 20 patients with Fabry’s (half with LVH) and 10 control patients, those with Fabry’s showed reduced contraction and relaxation even before the development of LVH. Lateral or septal systolic velocities (Sa) < 10 cm/s or early diastolic velocities (Ea) < 10 cm/s each showed a sensitivity and specificity of 100% in mutation-positive patients without LVH.^46^

A recent larger study confirmed the usefulness of TDI in detecting sub-clinical cardiac involvement, showing significantly lower Ea velocities (lateral 12.00 ± 3.34 cm/s, septal 5.52 ± 2.12 cm/s), although Sa velocities did not differ between Fabry’s patients and controls. Late diastolic velocities (Aa) and isovolumic contraction times (IVCT) were also significantly lower in Fabry’s patients without LVH compared to controls. Isovolumic relaxation times (IVRT) were significantly longer in all Fabry’s patients compared to controls. IVCT ≤ 105 ms was the best predictor for sub-clinical involvement with a sensitivity of 100% and specificity of 91%. An Ea velocity of < 15.5 cm/s had a sensitivity of 44.4% and specificity of 93.2% while IVRT > 60 ms had a sensitivity and specificity of 27.8 and 96.6%, respectively, for detecting pre-clinical cardiac involvement in Fabry’s patients.^42^

Therefore TDI may be a valuable and inexpensive modality for detecting cardiac involvement in hemizygous men and heterozygous or ‘carrier’ females.^28^

## Cardiac MRI

Cardiac MRI (CMR) has firmly established its usefulness in the evaluation of left ventricular dysfunction and cardiomyopathy, specifically in the absence of coronary artery disease on angiogram, i.e. non-ischaemic cardiomyopathy.^47^ CMR is non-invasive and can be used to not only assess cardiac function, but also for tissue abnormalities such as fibrosis, infiltration and inflammation.

With respect to Fabry’s cardiomyopathy, most commonly seen on CMR is regional or global myocardial hypertrophy; however, one potential distinguishing feature of cardiac hypertrophy due to Fabry’s disease is late enhancement of gadolinium, also called delayed contrast enhancement. Gadolinium enhancement occurs when chelated gadolinium stays in the intercellular space; in conditions causing fibrosis, the intercellular space is increased and distribution is slower.^48^ Areas of late gadolinium enhancement (LGE) in Fabry’s cardiomyopathy correspond histologically to collagen, but unlike post myocardial infarction scarring, the collagen is not in disarray.^49^ The scarring in Fabry’s is not as well defined as fibrosis seen post myocardial infarction (MI), with LGE being able to differentiate between the two.^47^

Although LGE is not a specific finding with regard to restrictive or hypertrophic cardiomyopathies and can be seen in any aetiology of LVH,^50^ fibrosis may be more focal than other forms of cardiomyopathy (e.g. versus global sub-endocardial involvement in amyloidosis) and various cardiomyopathies may have different tissue predilections (e.g. papillary muscle in cardiac sarcoidosis).^51^ Although reports are not always consistent for various cardiomyopathies^52^ and more work needs to be done to evaluate the utility of LGE in differentiating different cardiomyopathies, 53 for reasons which have not been completely elucidated, the earliest evidence of LGE in Fabry’s disease is the basal infero-lateral wall (also known as the postero-lateral wall).^48^,^54^-^56^

A recent study comparing patients with symmetric hypertrophic cardiomyopathy and Fabry’s cardiomyopathy found late gadolinium enhancement of the infero-lateral basal or mid-basal segments sparing the sub-endocardium to be specific for Fabry’s.^57^ However, LGE is only present in severe stages of Fabry’s cardiomyopathy, reflecting the extensive fibrosis due to glycosphingolipid accumulation,^55^,^56^ and LGE has been shown to correlate with a poorer prognosis in non-ischaemic cardiomyopathy.^58^

Another potential method for screening patients with Fabry’s disease for early cardiac involvement or differentiating physiologic LVH from Fabry’s cardiomyopathy using CMR is T2 relaxation time, which has been shown to be prolonged in Fabry’s patients with and without increased myocardial wall thickness.^59^

## ECG

ECG findings are non-specific but may show manifestations of conduction tissue infiltration, such as PR prolongation,^38^ varying degrees of heart block, sinus bradycardia, sick sinus syndrome, and atrial or ventricular arrhythmias.^39^ Myocardial infiltration may present as evidence of atrial enlargement or LVH.

## Screening patients with LVH for Fabry’s disease

Of patients with late-onset cardiac hypertrophy, the prevalence of Fabry’s disease has been reported to be as high as 6% in men (mean age 53 years)^60^ and 12% in women (mean age 50 years).^61^ A recent study from Spain showed the incidence to be 1% by genotyping (mean age 58 years) (0.9% in men and 1.1% in women), although low alpha-galactosidase activity was present in 3%.^62^ Strong consideration should be made to check alphagalactosidase A activity in middle-aged patients with hypertrophy in the absence of long-standing hypertension.

## Treatment

Enzyme replacement therapy (ERT) for the treatment of Fabry’s disease was first performed in the 1970s,^63^ however, open-label phase 2 trials were not performed until the 2000s.^63^ ERT using recombinant human alpha-galactocidase A (generic names agalsidase alpha and agalsidase beta) was approved for use in Europe in 2001 and in the United States in 2003.^64^

Initial randomised controlled trials (RCT) showed that 69% of the treatment group was free of renal microvascular endothelial deposits of globotriaosylceramide (primary endpoint) versus no change in the placebo group after 20 weeks (*p* < 0.001). There was also a statistically significant difference in endothelial deposits in the heart (*p* < 0.001).^63^

Although there did not appear to be a difference in quality of life as assessed by the SF-36,^63^ another RCT showed a statistically significant decrease in pain severity and improvement in quality of life (primary outcome). This study also showed improvement in renal architectural distortion (mesangial diameter) (*p* < 0.01) and increase in creatinine clearance (*p* < 0.02).^65^

In a more recent, larger RCT, 42% of placebo patients versus 27% of treated patients had clinical events (defined as renal, cardiac or cerebrovascular event or death); the time to first clinical event adjusted for baseline proteinuria favoured agalsidase beta but included the null (hazard ratio 0.47, CI: 0.21–1.03; *p* < 0.06). Time to first cardiac event (arrhythmia, angina or MI) was not affected. Although overall the results were less than overwhelming, treatment effect was greater in patients with preserved renal function.^64^ There are no data currently regarding ERT and affect on mortality.

## Cardiomyopathy

Observational studies have been performed which specifically analyse cardiac endpoints in Fabry’s patients on ERT. Several studies have documented statistically significant improvement in LVH by echocardiography^31^,^66^ and CMR.^67^,^68^ Hughes *et al.* also document a 20% reduction in myocardial Gb_3_ content by cardiac biopsy at six months of therapy, versus a 10% increase in patients receiving placebo.^68^ At least one study has documented an improvement in diastolic dysfunction (29% decrease of E/Ea; *p* < 0.002), although there was no improvement in LVH or renal function.^69^ However, at two years, one study has shown no statistically significant changes on ECG, stress echocardiography or CMR, although one-year follow-up data did look promising.^70^

Although improvement in symptoms of CHF and angina have been reported as improved with therapy,^31^ this remains open to question.^70^ The lack of efficacy may be due to the degree of fibrosis. As hypothesised previously,^48^ a recent case-control study found a statistically significant reduction in LVH (*p* < 0.001), improved myocardial function by TDI (*p* = 0.045) and improved exercise capacity (*p* = 0.014) in patients with no fibrosis by late gadolinium enhancement at three years. However, in patients with mild or severe fibrosis, there was only minimal improvement in LVH and no improvement in LV function or exercise capacity.^71^

## Angina and arrhythmias

Interestingly, in one series, conduction and valvular abnormalities were more common in patients receiving treatment, most likely reflecting the biases of observational studies.^31^ Overall, there is little data on the incidence/prevalence of arrhythmias with and without treatment, however one author reports on the lengthening of an ‘abbreviated PR interval’ in one patient.^72^ Coronary microvascular dysfunction does not appear to improve on therapy,^73^,^74^ nor does angina improve despite improvement in LVH,^70^,^73^ however, this may be confounded by the degree of cardiac involvement (see above). Regardless, this does imply that angina may be more related to microvascular dysfunction than to hypertrophy and supply–demand mismatch of oxygenation.

## Conventional therapy

Fabry’s patients with obstructive coronary artery disease should be managed as any other patient, including treatment with aspirin, lipid-lowering therapy, anti-anginals, beta-blockers, etc, and revascularisation should be according to the standard of care.^75^ However, special considerations with regard to coronary artery bypass grafting may be warranted. One case report has suggested that at least in untreated Fabry’s patients, vein grafts may be better conduit vessels than the left internal mammary artery due to an increased amount of sphingolipid infiltration, thus making arterial conduits more susceptible to premature failure.^76^ Significant valvular disease should be managed per the current guidelines,^77^ and case reports of both aortic^78^ and mitral^79^ valve replacements have been documented. Pacemaker for symptomatic bradycardia, heart block, etc. should be implanted according to current device guidelines.^80^

Medical therapy for Fabry’s patients with systolic dysfunction should include: angiotensin-converting enzyme inhibitor (ACEI) or aldosterone receptor blocker (ARB), beta-blocker, hydralazine plus nitrate in patients of African descent after maximal titration of ACEI or ARB, and diuretics for volume management. Furthermore, ICD and biventricular pacing should also be considered after maximal medical therapy as per guidelines.^81^ Diastolic dysfunction should be treated with diuretics for symptom management, although there is some retrospective data that statins may improve mortality.^82^

Symptomatic Fabry’s cardiomyopathy mimicking hypertrophic obstructive cardiomyopathy may benefit from septal alcohol ablation.^83^ Fabry’s patients with end-stage heart failure on maximal medical therapy should also be considered for transplant regardless of the aetiology.^84^ Interestingly, the transplanted organ would also produce alpha-galactosidase A, possibly protecting from future cardiac abnormalities due to glycosphingolipid accumulation.

Based on limited data from small studies, it appears that treatment should be initiated early, ideally before the development of fibrosis (late enhancement on CMR).^71^ However, ERT does not replace conventional medical therapy for arrhythmias, angina and heart failure.

## Conclusion

Although Fabry’s disease in the general population is rare, it may be relatively common in patient’s presenting with late cardiac hypertrophy. Cardiac involvement in both the classical and cardiac variant, Fabry’s disease is characterised by arrhythmias and LV hypertrophy. The hypertrophy in Fabry’s cardiomyopathy can potentially be distinguished from other aetiologies by TDI when isovolumic contraction time is ≤ 105 ms and Ea is < 10 cm/s, and by expert interpretation of late gadolinium enhancement and prolonged T2 relaxation time on cardiac MRI. TDI may also be able to detect cardiac involvement before the development of hypertrophy.

Treatment with enzyme replacement therapy may decrease the frequency of cardiac events, decrease hypertrophy, and, if started early before the development of fibrosis, may improve cardiac function and prevent deterioration in functional capacity. Middle-aged patients presenting with hypertrophy, particularly in the absence of other common aetiologies, should be evaluated for alpha-galactosidase deficiency.
